# A Case of Large Solitary Fibrous Tumor in the Retroperitoneum

**DOI:** 10.4137/ccrep.s2356

**Published:** 2009-03-23

**Authors:** Takeo Nomura, Ryuta Satoh, Kenji Kashima, Mutsushi Yamasaki, Kenichi Hirai, Fuminori Satoh, Hiromitsu Mimata

**Affiliations:** 1 Department of Oncological Science (Urology), Oita University Faculty of Medicine, 1-1 Idaigaoka, Hasama-machi, Yufu, Oita 879-5593, Japan.; 2 Pathology Center, Oita University Faculty of Medicine, 1-1 Idaigaoka, Hasama-machi, Yufu, Oita 879-5593, Japan.

**Keywords:** solitary fibrous tumor, retroperitoneum, latissimus dorsi muscle

## Abstract

Solitary fibrous tumor (SFT) is a rare spindle cell neoplasm mainly originated in the pleural cavity. We report here an unusual case of a large SFT in the retroperitoneum. A 27-year-old female complaining of a palpable mass in the right flank with dull pain was admitted to our hospital with the diagnosis of right retroperitoneal tumor. Computed tomography (CT) and magnetic resonance imaging (MRI) showed a large retroperitoneal tumor arising from latissimus dorsi muscle. Surgical findings revealed a partly encapsulated tumor and complete resection of tumor concomitantly with right kidney, 11th and 12th ribs, and diaphragm was performed. Pathological examination demonstrated the tumor to be composed of increased mitotic activity and cellularity of spindle cells with a collagenous matrix. Immunohistochemical staining was positive for CD34, vimentin, and basic fibroblast growth factor (bFGF) and negative for CD31, cytokeratin, desmin, S-100p, smooth muscle actin, Bcl-2, and insulin-like growth factor (IGF) with Ki-67 labeling index of 0.1%. Based on pathological features, diagnosis of SFT in the retroperitoneum was confirmed. To our knowledge, this is the first report of an SFT arising from latissimus dorsi muscle and it is important to include SFT in the differential diagnosis of retroperitoneal tumors that caused considerable diagnostic problems due to its unusual site of origin.

## Introduction

Solitary fibrous tumor (SFT) is a mesenchyme-derived tumor developed principally in the pleura and rarely is the extrapleural sites of origin.^1^ Recently, sporadic case reports of SFT in various extrapleural sites, such as the mediastinum,^2^ pericardium,^3^ nasal cavity,^4^ liver,^5^ renal capsule,^6^ thyroid gland,^7^ salivary gland,^8^ orbit,^9^ peritoneum,^10^ and retroperitoneum^11^ have appeared. Although the majority of SFT are benign, SFT has the potential for the malignant either clinically or histologically.^12^ The clinical behavior of SFT is notoriously unpredictable because of the histological variety and rarity. Immunohistochemical analysis, therefore, is of adjunctive importance in making the differential diagnosis and predicting the behavior of this disease. We describe an unusual case of large retroperitoneal SFT arising from latissimus dorsi muscle.

## Case Report

**Figure 1:**
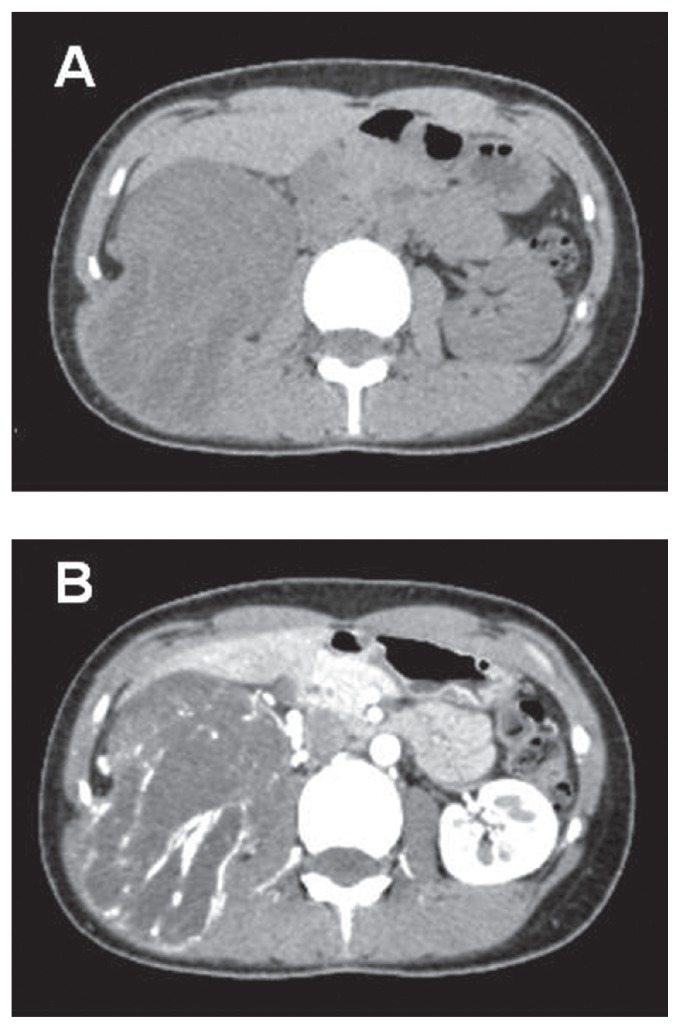
**A**) Abdominal computed tomography demonstrated the presence of a solid encapsulated mass with nonuniform internal structure about 11 × 9 cm in diameter, compressing the right kidney to the caudal side; **B**) contrast-enhanced CT showed a tumor with early enhancement in mainly dorsal side of the tumor.

**Figure 2:**
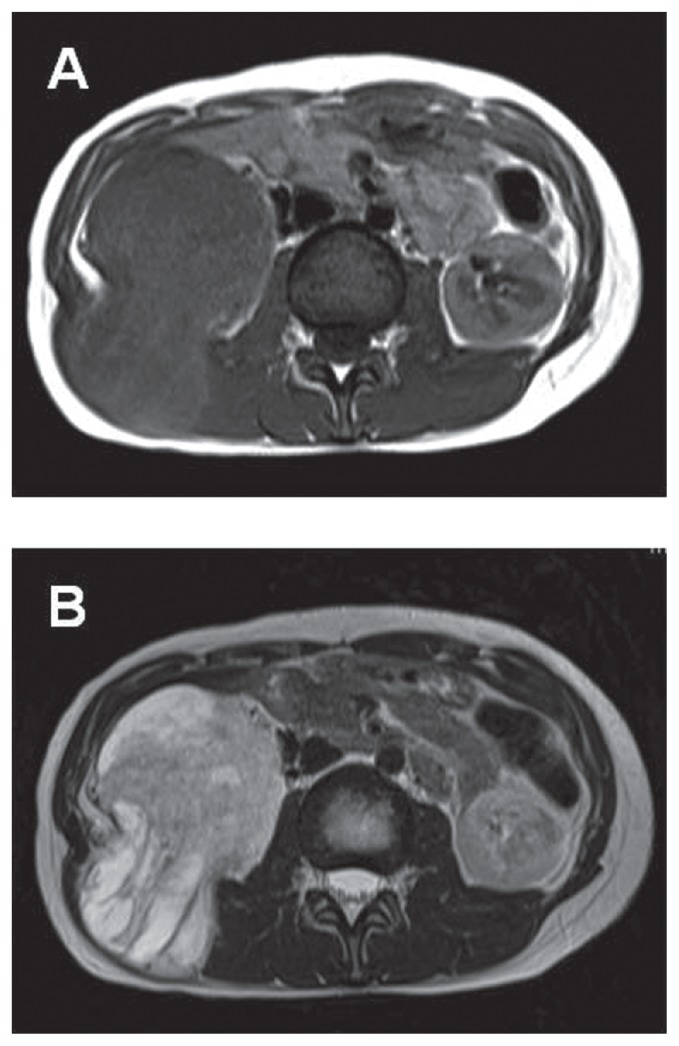
**A**) Magnetic resonance imaging demonstrated a tumor that had relatively smooth margin in the retoperitoneum exhibiting mainly high intensity on T1 weighting; **B**) T2-weighted image showed a high signal intensity tumor with low signal intensity area inside.

A 27-year-old female noticed a palpable swelling in the right flank with dull pain, and was referred to our hospital with the diagnosis of right retroperitoneal tumor. On physical examination, an elastic hard fixed tumor occupying the right abdomen was palpable. Abdominal computed tomography (CT) demonstrated the presence of a solid encapsulated mass with nonuniform internal structure about 11 × 9 cm in diameter, compressing the right kidney to the caudal side (Fig. 1a). Contrast-enhanced CT showed a tumor with some early staining in mainly dorsal side of the tumor, suggesting that the tumor feeding was supplied by the feeders from lumbar muscles such as latissimus dorsi muscle, quadratus lumbar muscle, and psoas major muscle (Fig. 1b). On magnetic resonance imaging (MRI), the large tumor having relatively smooth margin in the retoperitoneum exhibited mainly high intensity on both T1 (Fig. 2a) and T2 weighting (Fig. 2b). Neither lymph node swelling nor distant metastasis were detected by chest X-ray and CT. Based on these results, we diagnosed a retroperitoneal tumor arising from latissimus dorsi muscle.

**Figure 3:**
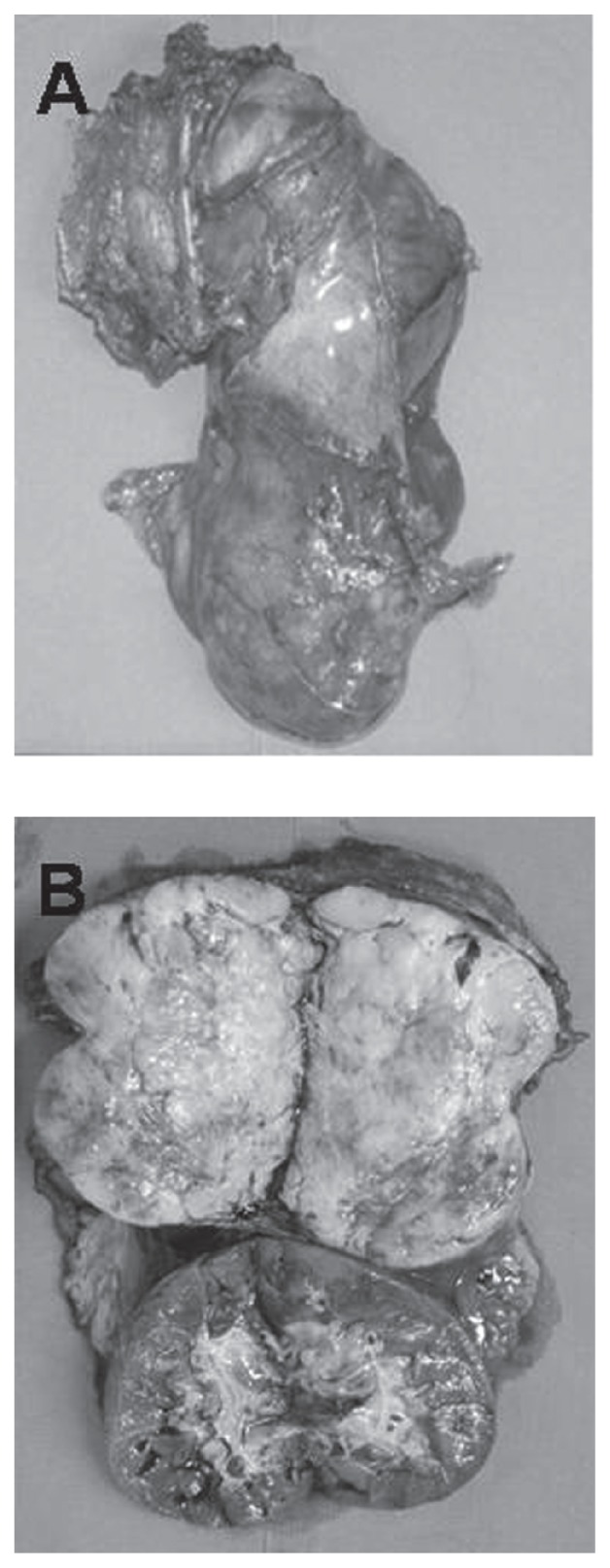
Surgical specimen: **A**) Macroscopic findings showed a well-circumscribed and encapsulated elastic hard tumor, 12 × 10 × 9 cm in diameter; **B**) Cross section showed yellowish fibrous lobulated solid mass with a whorled or nodular appearance.

**Figure 4:**
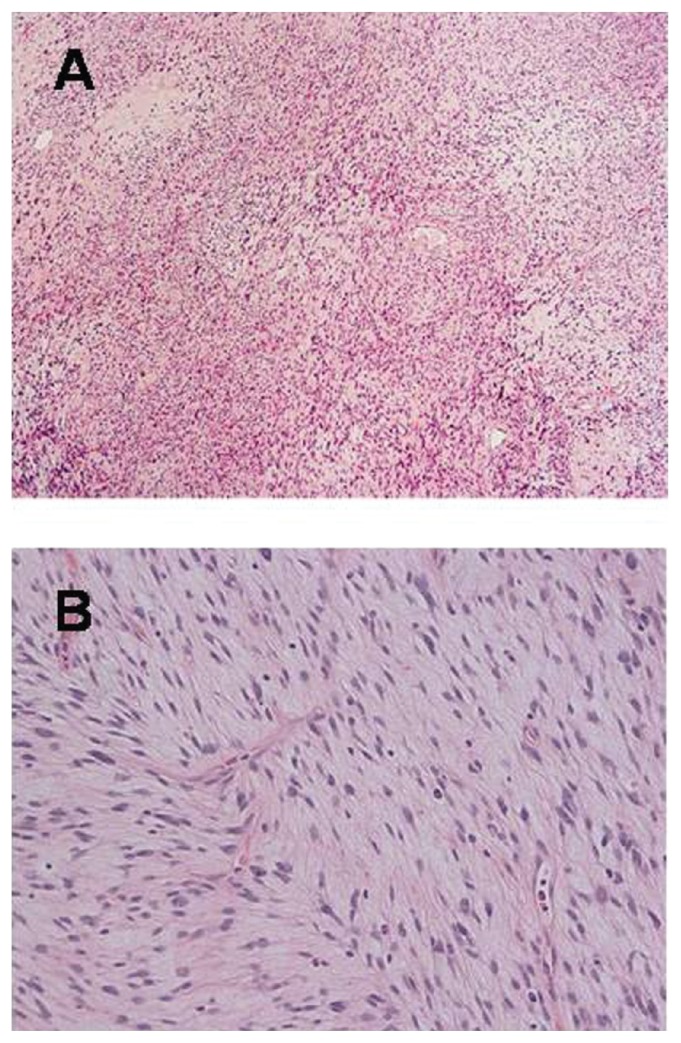
Histological findings (H&E staining): **A)** The tumor consisted of spindle-shaped cells with patternless pattern and hemangiopericytomatous appearance (×40); **B**) The tumor cells had oval or fusiform nuclei with fine chromatin and indistinct cytoplasm (×200).

**Figure 5:**
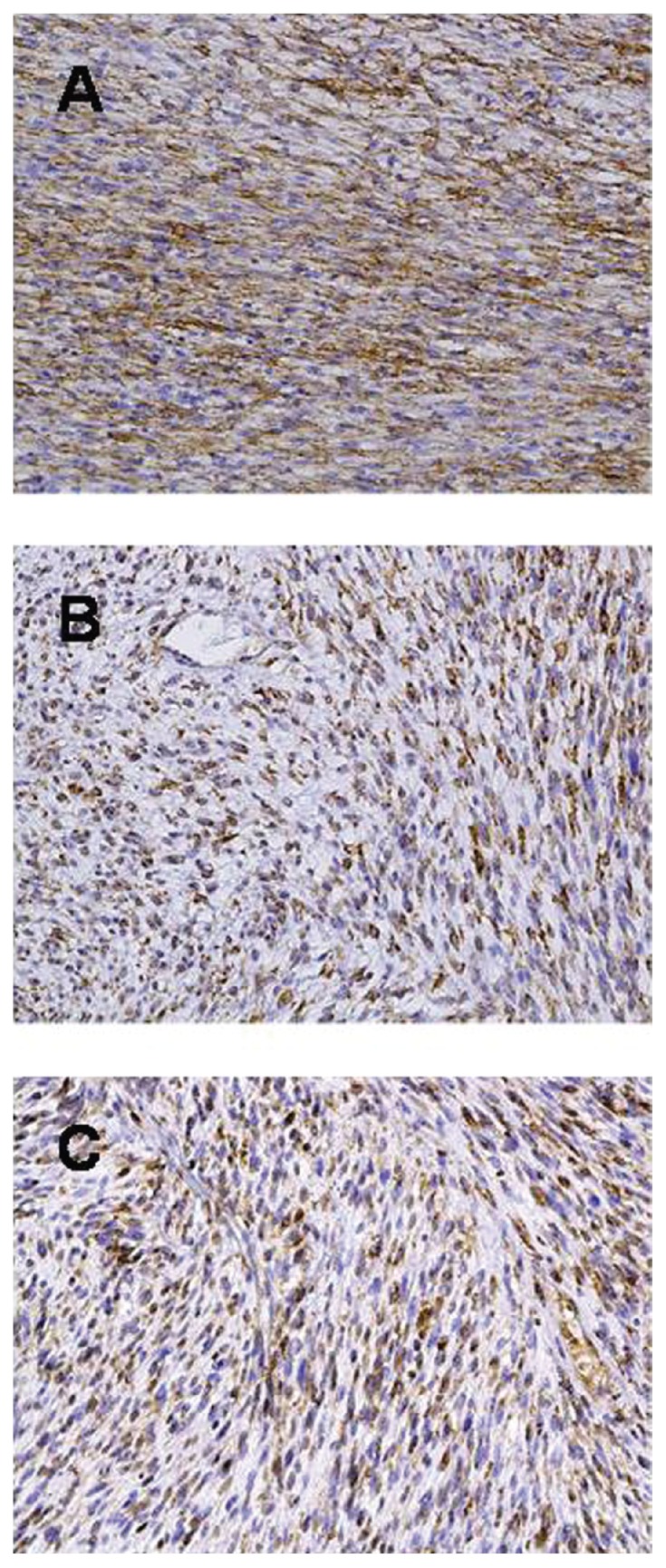
Immunohistochemical staining: **A**) CD34; **B**) vimentin; **C**) bFGF.

At operation, a smooth-surfaced large tumor occupied the right retroperitoneal cavity compressing the right kidney to the caudal side. Most of the tumor was encapsulated and easily mobilized using a blunt and sharp dissection along the tumor capsule. Because the tumor showed infiltrative growth to the latissimus dorsi muscle and 11th and 12th ribs, tumor extirpation with removal of the right kidney, 11th and 12th ribs, and part of diaphragm was performed. Macroscopic findings showed a well-circumscribed and encapsulated elastic hard tumor, 12 × 10 × 9 cm in diameter, with partly infiltrative growth into latissimus dorsi muscle (Fig. 3a). Cross section of specimens showed yellowish fibrous lobulated solid mass with a whorled or nodular appearance (Fig. 3b). Pathological examination revealed that the tumor was composed of spindle-shaped cells with varied cellular intensity, consisting of a mixture of haphazard or interlacing fascicular arrangements of spindle-shaped cells and collagenous matrix with patternless pattern and hemangiopericytomatous appearance (Fig. 4a). And the tumor cells were randomly arranged with abundant necrotic areas. The tumor cells had oval or fusiform nuclei with fine chromatin and indistinct cytoplasm (Fig. 4b). Invasion into the renal capsule, 11th and 12th ribs, diaphragm, and skin were not observed. Immunohistochemical staining was positive for CD34 (Fig. 5a), vimentin (Fig. 5b), and basic fibroblast growth factor (bFGF) (Fig. 5c) and negative for CD31, cytokeratin, desmin, S-100p, smooth muscle actin, Bcl-2, and insulin-like growth factor (IGF) with Ki-67 labeling index of 0.1%, confirming the diagnosis of SFT in the retroperitoneum. Based on the operative findings, we suspected that this retroperitoneal tumor originated in the latissimus dorsi muscle, but this could not be proven pathologically. The patient had an uneventful postoperative course and was discharged 9 days after her operation. No further treatment was performed, and she has no signs of recurrence for 6 months after surgery.

## Discussion

SFT is rather rare, an uncommon soft tissue tumor that usually develops in the pleura.^1^ According to previous studies, approximately 30% of SFT arise in extrapleural locations.^1^ The occurrence of extrapleural SFT has been occasionally reported with the most common unusual sites being the mediastinum,^2^ pericardium,^3^ nasal cavity,^4^ liver,^5^ renal capsule,^6^ thyroid gland,^7^ salivary gland,^8^ orbit,^9^ peritoneum,^10^ and retroperitoneum.^11^ SFT was initially thought to be of mesothelium origin and therefore termed solitary/localized mesothelioma,^13^ but its histogenesis has been controversial. Recent histological and immunohistochemical studies have defined fibrous tumors in this category as solitary/localized fibrous tumors derived from ubiquitous dendritic interstitial cells such as submesothelial stromal cell or fibroblast like mesenchymal cell.^14^ The histological characteristics of SFT were the so-called “patternless pattern” characterized by a haphazard, storiform arrangement of spindle cells, and “hemangiopericytoma-like appearance” with prominent vascularity.^15^ Indeed the possibility exists that the tumor originated from retroperitoneum extended to muscle, but we were careful to point out during the operation that the primary tumor in this patient originated in the latissimus dorsi muscle and extended to the retroperitoneal space because the tumor feeding was supplied by the feeders from lumbar muscles and half of the tumor located in the latissimus dorsi muscle. This anatomic origin of SFT has not yet been reported, and then to establish a preoperative diagnosis was impossible because SFT in the retroperitoneum is rare. Therefore, immunohistochemical staining is helpful for establishing the diagnosis, in particular CD34, which is considered as a positive marker for SFT.^16^ CD34 is expressed on the surface of lymphohematopoietic stem and progenitor cells, small-vessel endothelial cells, and embryonic fibroblasts.^17^ In addition to the expression of CD34, the positive finding for vimentin, a general marker of cells originating in the mesenchyme, seems to support the diagnosis of SFT. Other similar spindle cell neoplasms mixed with adipose tissue, such as dendritic fibromyxolipoma, lipomatous hemangiopericytoma, and spindle cell lipoma, should be distinguished from SFT. The histological and immunohistochemical features are helpful for excluding other CD34-positive tumors, such as neural tumors and smooth muscle tumors. Immunoreactivity of the present tumor for CD34 and vimentin, coupled with the absence of immunoreactivity for CD31, cytokeratin, desmin, and S-100p, strongly favored a definite diagnosis of SFT. Although the retroperitoneal tumors may remain clinically silent until they are advanced, SFT elicits the symptom of hypoglycemia attributed to IGF produced by tumors.^18^ Our patient showed no signs of hypoglycemia and negative finding for IGF staining was observed. Because the behavior of SFT is currently unpredictable and morphological distinction between benign and malignant SFT is often difficult, staining against Bcl-2, Ki-67 and bFGF may help for predicting the malignant potential of this tumor. Hasegawa et al. reported that 75% of extrapleural SFT showed positive reactivity for Bcl-2,^19^ which suppresses apoptosis, supporting the view that the expression of Bcl-2 can be used together with CD34 in the diagnosis of SFT. In addition, Sun et al. reported that Ki-67 and bFGF are relevant to the malignant potential of SFT and these markers are potentially useful for predicting prognosis of SFT.^20^ Ki-67 is known as a tumor proliferation marker, which is expressed in G1, S, G2, and M phases as assessed by cell-cycle analysis.^21^ Basic fibroblast growth factor is a member of a family of growth factors that stimulate proliferation of cells of mesenchymal, epithelial and neuroectodermal origin, promoting angiogenesis and mitogenesis.^22^ These markers are believed to play a role in differentiation of benign and malignant phenotype of SFT, suggesting the usefulness of immunoreactivity in the diagnosis of clinically malignant SFT. Our patient was negative for Bcl-2 with low Ki-67 labeling index of 0.1%, but positive for bFGF, suggesting potentially malignant tumor. In addition, it has been reported that some clinically aggressive SFTs with local recurrence and distant metastasis had low Ki-67 index (<10%).^19^ Thus, the clinical behavior of SFT is rarely so clear in individual cases and not always predictable from the histological and immunohistochemical findings even in the absence of atypical features. The most important prognostic factor in SFT seems not to be the histological appearance but to be the resectability.^19^ Therefore, complete surgery should be the treatment of choice if the lesion is resectable, and radical excision of the tumor merely carries the best prognosis. Preoperative correct diagnosis is seldom achieved and aggressive surgical management should always be the first choice both because of diagnostic uncertainty and of the possibility of a highly malignant SFT, as in the present case.

In summary, we describe an unusual case of large retroperitoneal SFT arising from latissimus dorsi muscle. The possibility of an extrapleural location of SFT must be kept in mind to set up the correct diagnosis, because symptoms and radiologic findings do not lead support to a diagnosis of retroperitoneal SFT. Most extrapleural SFTs usually have a favorable prognosis after complete local excision, but may have a potential to recur or metastasize. For this reason, careful clinical long-term follow-up is necessary for all patients with SFT.
